# Bile Acid Regulates the Colonization and Dissemination of *Candida albicans* from the Gastrointestinal Tract by Controlling Host Defense System and Microbiota

**DOI:** 10.3390/jof7121030

**Published:** 2021-11-30

**Authors:** Shankar Thangamani, Ross Monasky, Jung Keun Lee, Vijay Antharam, Harm HogenEsch, Tony R. Hazbun, Yan Jin, Haiwei Gu, Grace L. Guo

**Affiliations:** 1 Department of Comparative Pathobiology, College of Veterinary Medicine, Purdue University, West Lafayette, IN 47906, USA; hogenesc@purdue.edu; 2 Purdue Institute for Immunology, Inflammation and Infectious Diseases (PI4D), West Lafayette, IN 47906, USA; 3 College of Veterinary Medicine, Midwestern University, Glendale, AZ 85308, USA; rmonas@midwestern.edu (R.M.); jlee2@midwestern.edu (J.K.L.); 4 Department of Chemistry, College of Arts, Humanities and Sciences, Methodist University, Fayetteville, NC 28311, USA; vantharam@methodist.edu; 5 Department of Medicinal Chemistry and Molecular Pharmacology, College of Pharmacy, Purdue University, West Lafayette, IN 47906, USA; thazbun@purdue.edu; 6 Arizona Metabolomics Laboratory, College of Health Solutions, Arizona State University, Phoenix, AZ 85004, USA; kimyeon909@gmail.com (Y.J.); hgu@fiu.edu (H.G.); 7 Center for Translational Science, Department of Environmental Health Sciences, Robert Stempel College of Public Health and Social Work, Florida International University, Port St. Lucie, FL 33199, USA; 8 Department of Pharmacology and Toxicology, Earnest Mario School of Pharmacy, Rutgers University, Piscataway, NJ 08854, USA; glg48@eohsi.rutgers.edu; 9 Department of Veterans Affairs New Jersey Health Care System, East Orange, NJ 07018, USA

**Keywords:** bile acid metabolites, fungal colonization and dissemination, microbiota, host defense system

## Abstract

*Candida albicans* (CA), a commensal and opportunistic eukaryotic organism, frequently inhabits the gastrointestinal (GI) tract and causes life-threatening infections. Antibiotic-induced gut dysbiosis is a major risk factor for increased CA colonization and dissemination from the GI tract. We identified a significant increase of taurocholic acid (TCA), a major bile acid in antibiotic-treated mice susceptible to CA infection. In vivo findings indicate that administration of TCA through drinking water is sufficient to induce colonization and dissemination of CA in wild-type and immunosuppressed mice. Treatment with TCA significantly reduced mRNA expression of immune genes ang4 and Cxcr3 in the colon. In addition, TCA significantly decreased the relative abundance of three culturable species of commensal bacteria, *Turicibacter sanguinis, Lactobacillus johnsonii*, and *Clostridium celatum*, in both cecal contents and mucosal scrapings from the colon. Taken together, our results indicate that TCA promotes fungal colonization and dissemination of CA from the GI tract by controlling the host defense system and intestinal microbiota that play a critical role in regulating CA in the intestine.

## 1. Introduction

*Candida albicans* (CA), a commensal and opportunistic eukaryotic organism, frequently inhabits the gastrointestinal (GI) tract and can cause life-threatening infections [1,2,3,4]. CA is present in small numbers in the healthy GI tract of humans; thus, CA is harmless in immunocompetent humans [5,6]. While dysregulation in the host defense system contributes to invasive CA infections, the use of broad-spectrum antibiotics is a major predisposing risk factor for increased fungal colonization, and subsequent dissemination from the intestine [7,8,9,10,11,12,13,14]. CA is normally absent in the GI tract of adult mice; however, antibiotic treatment leads to CA colonization and dissemination in mice, and closely resembles CA infection in human patients [5,6,14,15,16,17,18,19,20,21,22]. Because colonization of the GI tract is necessary for the dissemination of CA, and CA colonization resistance is nullified by antibiotic treatment, identifying the factors that play a critical role in CA colonization may pave the way to develop novel approaches to limit CA dissemination [6,20].

Antibiotics alter the intestinal microbiota and lead to changes in the composition of the gut microbial metabolites that play a critical role in controlling several enteric bacterial pathogens [23,24,25]. However, there is limited knowledge about the potential role of microbial metabolites in the regulation of CA colonization and pathogenesis. To address this knowledge gap, we previously performed targeted metabolomics, and identified several groups of metabolites that are differentially regulated in the gut contents of cefoperazone-treated CA-susceptible mice and control CA-resistant mice [26]. A bile acid, taurocholic acid (TCA), was identified as one of the major classes of metabolites that was considerably increased in the antibiotic-treated mice [26]. Following synthesis in the liver, TCA enters the intestine and undergoes two major chemical modifications (*deconjugation and dihydroxylation*) carried out by gut microbes [27,28,29]. Antibiotic treatment depletes TCA-metabolizing commensal bacteria, leading to increased levels of TCA in the gut contents [26]. Furthermore, the gut concentration of TCA is considerably altered in different diseases. Specifically, TCA is highly up-regulated in immunocompromised cancer patients [30,31,32], drug-induced liver injury [33,34,35], high-fat diets [36], and liver cirrhosis [37,38,39,40,41,42], conditions that are associated with severe morbidity and mortality caused by CA. Numerous FDA-approved drugs and probiotics currently used to treat various diseases also profoundly alter bile acid levels, including TCA in the gut [43,44,45,46,47,48,49,50,51,52]. Therefore, understanding the role of TCA in the regulation of CA will gain insights into the bile-mediated regulation of gut fungi, and will form a platform to develop novel therapies to control and treat invasive CA infections that arise as a result of antibiotic-induced dysbiosis, and to control CA-associated infections in individuals with liver cirrhosis. Therefore, in this study, we investigated the role of TCA on CA colonization and dissemination from the intestine. Our findings indicate that TCA promotes fungal colonization and dissemination by altering the intestinal defense system and microbiota.

## 2. Materials and Methods

### 2.1. Strains and Reagents

*Candida albicans* SC5314 was provided by Dr. Andrew Koh at the University of Texas Southwestern Medical Center [6]. This study used Yeast Peptone Dextrose (YPD) (242810, BD Difco, Franklin Lakes, NJ, USA) agar (BP1423, Fisher, Waltham, MA, USA), as well as Bacteroides Bile Esculin Agar (BBE) (AS114, Anaerobe Systems, Morgan Hill, CA, USA) for various assays. Additionally, the antibiotics kanamycin (J61272, Alfa Aesar, Tewsbury, MA, USA), ampicillin (69-52-3, IBI Scientific, Dubuque, IA, USA), and streptomycin (S6501, Sigma-Aldrich, St. Louis, MO, USA) were used in media for fungal enumeration, and cefoperazone (J65185, Alfa Aesar, Tewsbury, MA, USA) was used in antibiotic pre-treatment. Taurocholic acid (TCA) (16215, Cayman Chemicals, Ann Arbor, MI) and cyclophosphamide (PHR1404, Millipore Sigma, St. Louis, MO, USA) were purchased for these studies. Additional kits and reagents used included: hydrogen sulfide gas assay (ZAN-5084, Cell Biolabs, San Diego, CA), fluorescein isothiocyanate-dextran (FITC-dextran) (FD4, Millipore Sigma, St. Louis, MO, USA), and sodium formate (A17813, Alfa Aesar).

### 2.2. Fungal Colonization and Dissemination in the Immunosuppressed Mouse Model

Studies specifically examining survival and dissemination of mice infected with *C. albicans* SC5314 under immunosuppressive conditions were performed with BALB/c mice. Male and female mice (age 8 to 12 weeks old) were treated with or without cefoperazone (0.5 mg/mL) via drinking water, and drinking water was replaced with fresh antibiotic water every two days. After 7 days, all mice were infected with *Candida albicans* SC5314 via oral gavage with 1–2 × 10^8^ CFU/mouse. Three days post-infection, all mice were treated with 150 mg of cyclophosphamide (cyclo) per kilogram of mouse body weight by intraperitoneal injection. A second and third dose of cyclophosphamide was given at 5- and 7-days post-infection, respectively. Mice were monitored for survival, and euthanized if mice were moribund. After euthanasia, cecal contents, liver, and kidneys were collected and processed for fungal burden to assess the fungal dissemination as described before [26]. Briefly, gut contents and tissue homogenates were serially diluted and plated onto YPD agar plates containing broad-spectrum antibiotics, and incubated at 30 °C for 24 h to determine the colony-forming units (CFU).

### 2.3. Effect of TCA on Fungal Colonization and Dissemination in Mice

Male and female wild-type C57B/6 mice were infected with *C. albicans* SC5314 via oral gavage with 1–2 × 10^8^ CFU/mouse. One group of mice was treated with 1% TCA in the drinking water, and the water was changed every day. Control groups received sterile water. Fecal contents were collected at the indicated time points to determine the fungal load in feces. Mice were monitored for survival, and cecal contents, liver, and kidneys were processed to determine the fungal load and dissemination as described before [26]. To determine the effect of TCA on fungal colonization and dissemination in the immunosuppressed mouse model, male and female BALB/cJ mice (age 10 to 12 weeks old) were infected as described above, and all mice were treated with 150 mg of cyclophosphamide (cyclo) per kilogram of mouse body weight through intraperitoneal injection. The second and third doses of cyclophosphamide were given again at 5- and 7-days post-infection, respectively. 

### 2.4. FITC-Dextran Permeability Assay

Gut permeability was measured in infected mice using a FITC-dextran assay. Wild type C57BL/6 mice were infected with *Candida albicans* SC5314 via oral gavage with 1–2 × 10^8^ CFU/mouse, and treated with or without 1% TCA through drinking water. After 10 days post-infection, mice were orally administered 150 uL PBS containing 15 mg FITC-dextran. The mice were anesthetized four hours later, and blood was collected via retro-orbital route. Blood samples were then centrifuged to collect serum. Serum samples were processed via a 2-fold serial dilution in a 96-well plate, and fluorescence was measured via plate reader (excitation: 485 nm; emission: 520 nm). Standard curves were made using serially diluted FITC-Dextran in PBS to determine the FITC-Dextran levels in serum samples. 

### 2.5. RNA Sequencing and Analysis

Wild type C57BL/6 mice were infected with *C. albicans* SC5314 via oral gavage with 1–2 × 10^8^ CFU/mouse, and treated with or without 1% TCA through drinking water. At 10 days post-infection, the colon was collected and flash-frozen in liquid nitrogen. Total RNA was collected using a Zymo Research RNA kit as per the manufacturer’s instructions. Total RNA-Seq libraries were constructed from 500 ng of total RNA, and rRNA was removed. Libraries were prepared using the Zymo-Seq RiboFree Total RNA Library Prep Kit (Cat # R3000) according to the manufacturer’s instructions. RNA-Seq libraries were sequenced on an Illumina NovaSeq to a sequencing depth of at least 30 million read pairs (150 bp paired-end sequencing) per sample. Sequence data alignments and differential expression analysis: NovaSeq paired-end 150-bp reads from Total RNA-Seq data files were first adaptor-trimmed, and then analyzed using the STAR program (version 2.6.1d) for alignment of short reads to the genome of interest. Transcript counts were inferred from alignment files. All transcripts with either 0 or 1 counts were removed. Gene expression was measured using EdgeR. The DESeq2 R package was used for differential expression analysis using the gene feature.

### 2.6. Microbiome Sequencing and Analysis

Wild type C57BL/6 mice were infected with *C. albicans* SC5314 via oral gavage with 1–2 × 10^8^ CFU/mouse, and treated with or without 1% TCA through drinking water. After 10 days post-infection, cecal contents and mucosal scrapings from the colon were collected using glass slides to determine the microbiome composition. Microbiome sequencing was carried out at Zymo Research corporation (Irvine, CA, USA). The ZymoBIOMICS^®^ Microbial Community Standard (Zymo Research, Irvine, CA, USA) was used as a positive control for each DNA extraction, if performed. The final library was sequenced on Illumina^®^ MiSeqTM with a v3 reagent kit (600 cycles), and the relative abundance of bacteria was analyzed as described elsewhere [26].

### 2.7. Immunofluorescence Staining

The colon from untreated and TCA-treated mice infected with CA were collected after 10–12 days of infection. Colon tissue was fixed in 10% neutral buffered formalin solution, sectioned at 5 µm, and stained with DAPI and ZO-1 Antibody (21773-1-AP, Thermofischer, Waltham, MA, USA). Stained tissues were imaged (40X) using a Keyence BZ-X700 microscope. 

### 2.8. Metabolomics

Bile acid metabolites (TCA and DCA) levels in the cecal contents were determined as described previously [26].

### 2.9. Statistical Analysis

Statistical analyses were performed using GraphPad Prism 6.0 (Graph Pad Software, La Jolla, CA). *p* values were calculated using the Mann–Whitney U test or unpaired Student’s *t*-test, or by one way-ANOVA followed by a Bonferroni comparison or Kaplan–Meier (log-rank) survival test as indicated. *p* values of (* ≤ 0.05), (** *p* ≤ 0.01), (*** *p* ≤ 0.001), and (**** *p* ≤ 0.0001) were considered as significant.

## 3. Results

### 3.1. TCA Is the Major Bile Acid Metabolite Up-Regulated in the Cefoperazone-Treated Mice Susceptible to CA Infection

**Figure 1:**
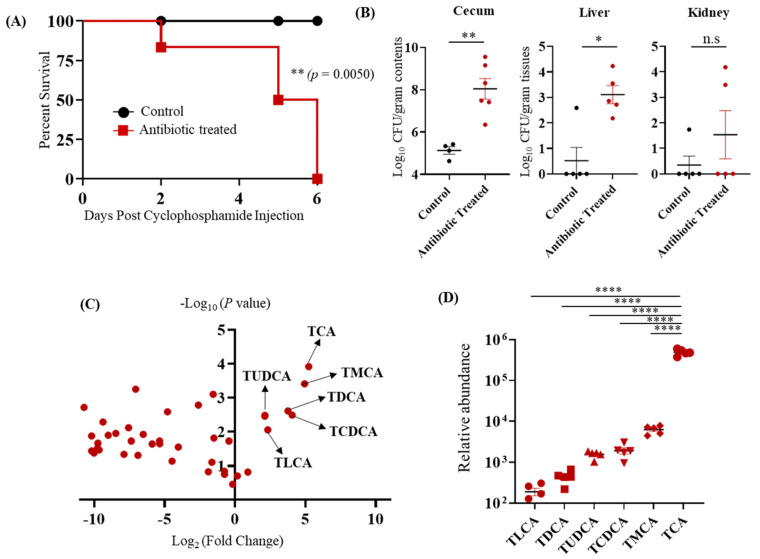
Taurocholic acid (TCA) is the major bile acid metabolite up-regulated in the cefoperazone-treated mice susceptible to CA infection. (**A**) BALB/c mice were fed sterile water in the presence or absence of cefoperazone (0.5 mg/mL) for 7 days and then infected with ~2 × 10^8^ CFU CA SC5314 via oral gavage. Antibiotic treatment was continued until the end of the experiment. Three days post-infection, all mice were injected with three doses of cyclophosphamide intraperitoneally (150 mg/kg body weight) and monitored for survival. A log-rank test was performed using 95% confidence intervals; statistical significance was calculated to compare the antibiotic-treated and untreated control groups. (**B**) Fungal load from cecum, liver, and kidney were collected immediately after death in the antibiotic-treated groups and mice euthanized after 6 days post-cyclophosphamide treatment from control groups. Statistical significance was evaluated using the Mann–Whitney U test. (**C**) Relative Log_2_ fold-change of bile acid metabolites in antibiotic-treated C57BL/6 mice relative to control groups. (**D**) Relative abundance of bile acid metabolites that were highly up-regulated and significant in the antibiotic-treated group relative to control groups. At least five mice per group were used, and the data represents mean ± SEM. one way-ANOVA followed by a multiple comparison using Bonferroni correction. *p* values of (* *p* ≤ 0.05), (** *p* ≤ 0.01), and (**** *p* ≤ 0.0001) were considered as significant.

To identify the metabolites that regulate CA colonization and dissemination, we treated BALB/c mice with or without a broad-spectrum antibiotic (cefoperazone), orally infected them with CA, and then injected them with cyclophosphamide intraperitoneally. As expected, all mice in the antibiotic-treated group died within 6 days of infection (Figure 1A). Furthermore, the fungal load was significantly higher in the cecal contents and liver of antibiotic-treated mice, thus, confirming increased CA colonization and dissemination from the GI tract of antibiotic-treated mice compared to control groups (Figure 1B). Previous findings from our lab indicate that more than 200 metabolites were differentially regulated in the cecal contents of control and antibiotic-treated mice [26]. Bile acids are one of the major metabolites differentially altered in the CA-susceptible mice compared to control groups [26]. Reanalysis of our previously published metabolomics data set indicates that six bile acids were significantly up-regulated in the antibiotic-treated CA susceptible mice (Figure 1C,D) [26]. Interestingly, only the abundance of TCA was significantly higher than all other bile acid metabolites that were increased in the antibiotic-treated CA susceptible mice compared to untreated control groups (Figure 1D). These results indicate that TCA is the highly up-regulated bile acid in the antibiotic-treated CA- susceptible mice compared to control groups. 

### 3.2. TCA Alone Induces Fungal Colonization and Dissemination from the GI Tract in the Absence of Antibiotics and Immunosuppressive Agents

**Figure 2:**
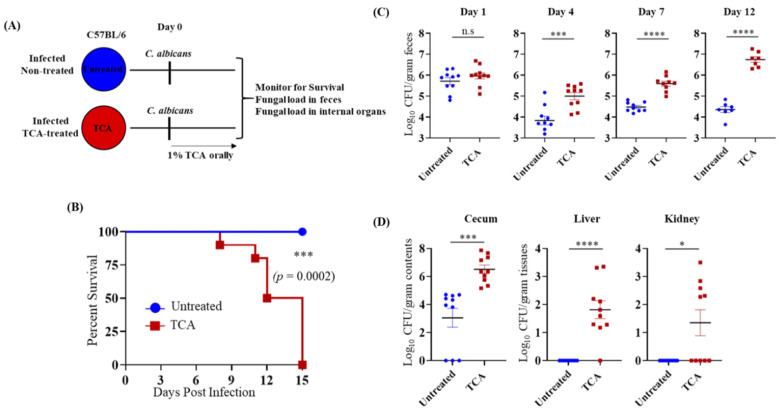
TCA alone induces fungal colonization and dissemination in the absence of antibiotics and immunosuppression. (**A**) Experimental outline. C57BL/6 mice infected with ~2 × 10^8^ CFU CA SC5314 via oral gavage. Untreated mice received sterile drinking water (untreated group); the treatment group received sterile water containing 1% TCA (TCA group). (**B**) Mice were monitored for survival. A log-rank test was performed using 95% confidence intervals; statistical significance was calculated to compare the antibiotic-treated and untreated control groups. (**C**) Fungal load from feces collected from untreated and TCA groups 1-, 4-, 7-, and 12-days post-infection. (**D**) Fungal load from cecum, liver, and kidney from dead mice was collected immediately in the TCA-treated groups and mice euthanized 15 days post-infection for untreated groups. Ten mice per group were used, and the data represent mean ± SEM. Statistical significance was evaluated using the Mann–Whitney U test. *p* values ≤0.05 (*), ≤0.001 (***), ≤0.0001 (****) were considered statistically significant.

To dissect the specific role of TCA in regulating CA colonization and dissemination in vivo, C57BL/6J mice infected with CA were treated orally with or without 1% TCA in the drinking water (Figure 2A). The fungal load in stool samples, and mortality were monitored. Mice treated with TCA alone died 15 days post-infection (Figure 2B).

Fungal load in the feces of the TCA group was significantly higher starting day 4 post-infection compared to the untreated control groups (Figure 2C). Furthermore, the TCA-treated group had significantly higher fungal loads in the cecal content, liver, and kidney, whereas no CA was detected in the liver and kidney from the untreated mice (Figure 2D). These results indicate that TCA alone can induce fungal colonization and dissemination from the GI tract in the absence of antibiotics and (or) immunosuppression.

### 3.3. TCA Induces Fungal Colonization and Dissemination from the GI Tract of Immunosuppressed Mice in the Absence of Antibiotic Treatment

**Figure 3:**
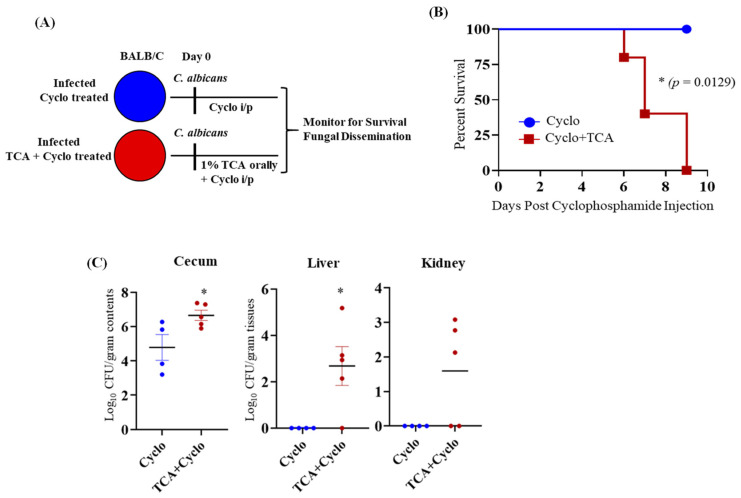
TCA induces fungal dissemination from the GI tract of immunosuppressed mice in the absence of antibiotic treatment. (**A**) Experimental outline. BALB/c mice infected with ~2 × 10^8^ CFU CA SC5314 via oral gavage received sterile water containing or not containing 1% TCA. Three days post-infection, all mice were injected with three doses of cyclophosphamide intraperitoneally (150 mg/kg body weight). (**B**) Mice were monitored for survival, and a log-rank test was performed using 95% confidence intervals; statistical significance was calculated to compare the antibiotic-treated and untreated control groups. (**C**) Fungal load from cecum, liver, and kidney collected immediately after death in the TCA+ cyclo-treated groups and mice euthanized 9 days post-infection for cyclo groups. 4–5 mice per group were used, and the data represent mean ± SEM. Statistical significance was evaluated using the Mann–Whitney U test. *p* values ≤ 0.05 (*) was considered statistically significant.

Since antibiotic-induced gut dysbiosis is necessary to induce CA colonization and dissemination in immunosuppressed mice (Figure 1A) [6], we tested whether administration of 1% TCA in the drinking water of immunosuppressed mice can promote fungal colonization and dissemination (Figure 3A). Interestingly, BALB/c mice that received TCA started to die 6 days post-cyclophosphamide injection, and all the mice were dead by day 9 after the first dose of cyclophosphamide. On the other hand, the mice that were infected with CA and received three doses of cyclophosphamide survived (Figure 3B). The fungal load was significantly increased in the cecal contents and liver of the TCA-treated group compared to the immunosuppressed mice that received only sterile drinking water (Figure 3C). These results suggest that an increased concentration of TCA in the intestine promotes fungal colonization, dissemination, and mortality, even in the absence of antibiotic treatment in immunosuppressed mice.

### 3.4. TCA Enhanced Intestinal Permeability and Reduced the Expression of a Tight Junction Protein 

**Figure 4:**
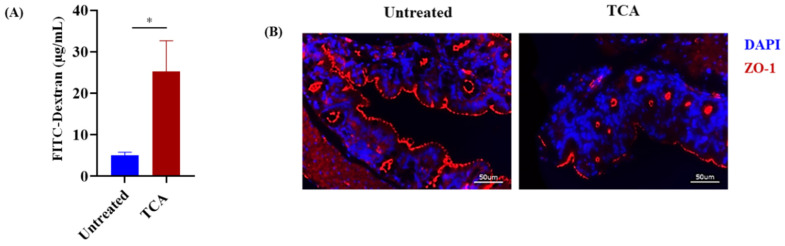
TCA increases intestinal permeability leading to fungal dissemination from the GI tract. (**A**) Gut permeability was measured in infected mice using a FITC-dextran assay. At 10 days post-infection, male and female BL57/6J mice in both control and TCA-treated groups infected with CA were given an oral gavage of 150 µL PBS containing 15 mg FITC-dextran. Four hours after administering FITC-dextran, mice were anesthetized, and blood was collected via retro-orbital bleed. Blood samples were processed via a two-fold serial dilution in a 96-well plate, and fluorescence was measured via a plate reader (excitation: 485 nm; emission: 520 nm). Data represent mean ± SEM. Statistical significance was evaluated using the Mann–Whitney U test. *p* values ≤ 0.05 (*) was considered statistically significant. (**B**) ZO-1 tight junction protein expression in untreated and TCA-treated mice. Colon tissue from untreated and TCA-treated mice from CA-infected mice (10 days post-infection) was stained with ZO-1 and DAPI antibodies. Representative images are shown here.

The intestinal barrier function is critical to prevent enteric pathogens from disseminating from the GI tract to systemic organs [6,53]. Since TCA treatment induced CA dissemination from the GI tract, we evaluated if TCA dysregulated the intestinal barrier function with a FITC-dextran permeability assay. The TCA-treated group had significantly increased levels of FITC-dextran in the blood compared to the untreated control groups (Figure 4A). Furthermore, the expression of the ZO-1 tight junction protein was decreased in the colon of TCA-treated mice (Figure 4B). Altogether, these results indicate that TCA dysregulates the intestinal barrier function, which may contribute to CA dissemination from the intestinal tract.

### 3.5. TCA Down-Regulates ang4 and Cx3cr1 Expression in the Colon Tissue

**Figure 5:**
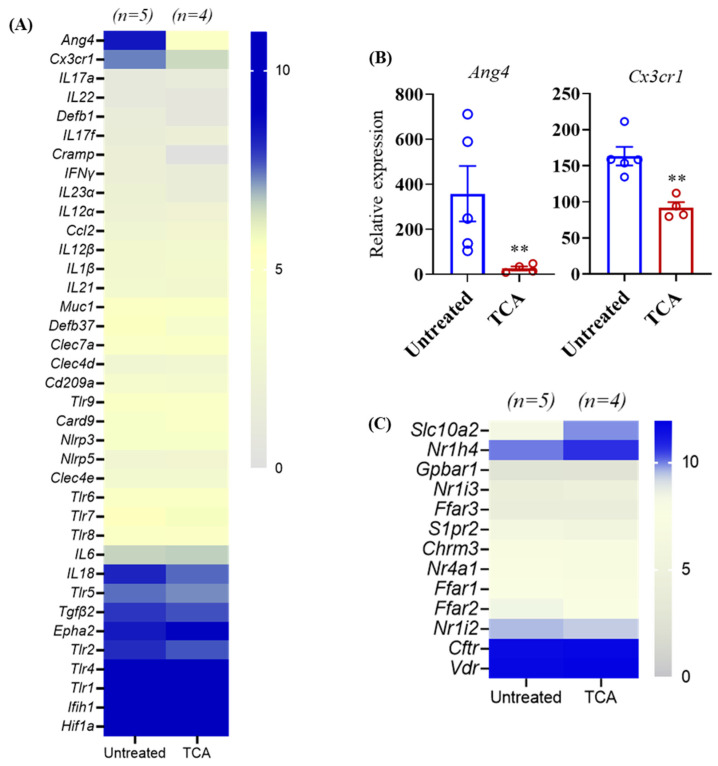
TCA inhibits the expression of *ang4* and CX3CR1 in the colon in vivo. Colon tissue from untreated and TCA-treated mice collected after 10 days of CA infection and treatment were RNA-sequenced to examine the expression level of host defense genes. (**A**) Average relative expression of host defense genes in untreated and TCA-treated mice is shown. (**B**) Relative expression of *ang4* and *Cx3cr1* in untreated and TCA-treated mice is shown. (**C**) Average relative expression of intestinal metabolite receptors in untreated and TCA-treated mice is shown. Four to five mice per group were used. Data represent mean ± SEM. Statistical significance was evaluated using the Student’s *t*-test. *p* values ≤ 0.01 (**) was considered statistically significant.

CA can cause severe invasive disease and mortality once critical components of the host defense system are compromised [6,53,54,55,56]. To identify if TCA dysregulates the intestinal host defense system to induce CA colonization and dissemination, colonic tissue from untreated and TCA-treated mice were collected after 10 days of CA infection, and were RNA-sequenced to examine the expression of host defense genes (Figure 5A). Among the host defense genes examined, the relative expression of angiogenin 4 (*ang4*), an antimicrobial peptide, and CX3CR1 (*Cx3cr1*), a chemokine receptor, was significantly down-regulated in TCA-treated mice compared to untreated mice infected with CA (Figure 5A,B). Bile acids receptors play an important role in bile acid signaling [50,57,58,59,60,61]. Further, our findings indicate that among the receptors examined, the relative expression of *Slc10a2* and *Nr1h4* that encode the apical sodium-dependent bile acid transporter (ASBT) and farnesoid X receptor (FXR) receptors, respectively, was increased in the TCA-treated mice compared to untreated groups infected with CA (Figure 5C).

### 3.6. TCA Alters Microbial Composition in Both Luminal and Mucosal Parts of the GI Tract

**Figure 6:**
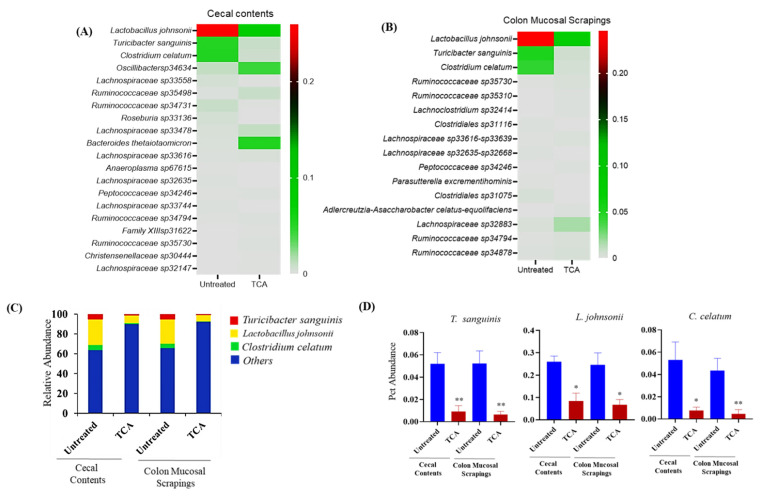
TCA alters the relative abundance of intestinal microbiota. C57BL/6 mice were infected with ~2 × 10^8^ CFU CA SC5314 via oral gavage. Control mice received sterile drinking water (untreated group); the treatment group received sterile water containing 1% TCA (TCA group). Ten days post-infection, cecal contents and colon scrapings were collected to determine the relative abundance of intestinal microbiota. (**A**,**B**) Bacterial members that are significantly altered in the TCA group compared to the untreated control groups in cecal content and colon mucosal scrapings, respectively, are shown here. (**C**,**D**) Bacterial members that are significantly altered in the TCA group compared to the untreated control groups in both cecal content and colon mucosal scrapings are shown here. Five mice per group were used. Data represent mean ± SEM. Statistical significance was evaluated using the Student’s *t*-test. *p* values ≤ 0.05 (*) or ≤ 0.01 (**) were considered statistically significant.

Next, we examined if TCA changes the composition of intestinal microbiota, as commensal bacteria are also essential in providing colonization resistance to CA [6,26]. We analyzed the intestinal microbiota composition from the cecal contents and mucosal scrapings from the colon in the untreated control and TCA-treated mice infected with CA. Our results revealed that several culturable and unculturable bacterial members were significantly altered in cecal contents and colon mucosal scrapings in both groups (Supplementary Figures S1–S4, Figure 6A,B). Out of the top 20 statistically significant culturable and unculturable bacterial members found in cecal contents and colon mucosal scrapings (Figure 6A,B), we focused on culturable bacterial members whose 16 s reads could be mapped at species-level resolution. The relative abundance of three culturable bacterial members: *Turicibacter sanguinis* (cecum: 5.2% versus 0.90%; colonic mucosa: 5.2% versus 0.65%); *Lactobacillus johnsonii* (cecum: 26.4% versus 8.0%; colonic mucosa: 24.6% versus 6.8%); and *Clostridium celatum* (cecum: 5.3% versus 0.76%; colonic mucosa: 4.3% versus 0.44%) were significantly decreased in both cecal content and colon scrapings from TCA-treated mice compared to untreated mice (Figure 6C,D). In mucosal scrapings, *L. johnsonii* constituted close to 25% of 16 s reads, but was three times more depleted in mice fed with TCA-infused drinking water. Comparatively, the diminishment as a result of TCA was 6- to 7-fold for reads mapping to *T. sanguinis* and *C. celatum*. Taken together, the relative abundance of three culturable bacterial members (*T. sanguinis*, *L. johnsonii*, and *C. celatum*) were significantly decreased in both cecal content and colon scrapings from TCA-treated mice compared to untreated mice infected with CA. 

## 4. Discussion

Our findings indicate that TCA is one of the most strongly up-regulated major bile acids in the antibiotic-treated mice susceptible to CA infection. After synthesis in the liver, TCA enters the small intestine and undergoes deconjugation and dihydroxylation, which are carried out by bile salt hydrolases and bile acid-inducible enzymes present in the gut microbes. These two chemical modifications convert TCA to DCA in the cecum [48,57,62,63,64,65,66,67]. Treatment with antibiotics decreases the microbial metabolism of TCA, and induces an increase of this bile acid. Treatment of wild-type and immunosuppressed mice with TCA alone promoted fungal colonization and dissemination from the GI tract in the absence of antibiotic treatment. Our studies revealed three primary mechanisms that underlie the TCA-induced enhanced CA colonization and dissemination from the GI tract, including: (i) deficiencies in host defense mechanisms; (ii) disruption of the commensal microbiota, which permits intestinal overgrowth of CA; and (iii) intestinal barrier dysfunction [6,53]. 

TCA significantly decreased the expression of *ang4* and *Cx3cr1* in the colon. Interestingly, both angiogenin-4 and CX3CR1 are involved in antifungal defense [55,68]. Mice have three orthologous *ang* antimicrobial peptide genes (*ang* 1, 3, and 4). However, only *ang4* is highly expressed in the colon and small intestine, and is produced by goblet and Paneth cells [68,69,70]. Humans only have one orthologous protein (ANG). Both *ang4* and ANG exhibit antifungal activity in vitro [68]. Similarly, CX3CR1, mainly expressed by macrophages in the intestine, plays a critical role in the antifungal defense to CA [55,71]. Furthermore, recent evidence indicates that CX3CR1-expressing macrophages are important to maintain intestinal barrier function to prevent bacterial translocation from the gut [72]. Mice dosed with 1% TCA orally had a significantly decreased abundance of *L. johnsonii, T. sanguinis*, and *C. celatum* in the cecal contents and colon mucosal scrapings. Interestingly, previous findings from our lab indicate that all these three bacterial genera were also decreased in antibiotic-treated mice susceptible to CA [26]. Commensal bacteria, such as *L. johnsonii*, play a critical role in maintaining the intestinal barrier function [73,74,75,76], and can directly inhibit the growth of fungi [77]. Since *ang4* and *Cx3cr1* expression is also regulated by the microbiota [68,78], it is possible that TCA indirectly regulates CA by controlling *ang4* and *Cx3cr1* expression through one of the three commensal bacteria modulated by TCA.

In the intestine, TCA is mainly absorbed through apical sodium-dependent bile acid transporter (ASBT), encoded by the *Slc10a2* gene in ileal and colonic epithelial cells [50,57,58]. After absorption, TCA binds to FXR, a nuclear receptor encoded by *Nr1h4*, expressed in enterocytes and macrophages, resulting in the transcription of target genes [50,57,59,60,61]. The expression of *Slc10a2* and *Nr1h4* was also significantly increased in the colon of antibiotic-treated mice [50,79]. Therefore, these findings, along with others [55,68,71,72], suggest that TCA may induce CA colonization and dissemination by controlling the intestinal defense system and barrier function through ASBT and FXR. Under normal physiological conditions, the intestine and cecum hold a bile salt concentration gradient ranging from 2% and 0.05% [80,81]. In addition, previous studies used 1% bile acids for mouse studies [82,83,84,85,86]. Therefore, we used an average concentration of 1% TCA in our experiments. However, the effect of TCA may differ depending on the intestinal concentration in the lumen. For example, a lower concentration (0.1 or 0.5%) may affect only microbiota or host, and can potentially enhance only CA colonization without dissemination. Further, in order to test the possibility that the metabolic transformation of orally administered TCA to DCA may contribute to host and microbiota alterations observed in our study, we also examined the DCA levels in TCA-treated mice. Surprisingly, mice that received TCA had considerably reduced levels of DCA compared to untreated groups (Supplementary Figure S5). This may be partly because TCA may directly inhibit certain species of bile-metabolizing bacteria, such as *Lactobacillus* species. 

Not only does antibiotic treatment pre-dispose the host to invasive CA infections, a recent study by Zhai et al. [4] indicates that individuals undergoing allogeneic hematopoietic cell transplantation (allo-HCT) were highly susceptible to CA and *Candida parapsilosis* invasive infections. Furthermore, the authors demonstrated that invasive fungal infections in allo-HCT patients originated from the intestinal tract [4]. The majority of primary bile acids in humans are conjugated to taurine (or) glycine, unlike mice, where the major conjugation is to taurine. Furthermore, antibiotic treatment in humans [87,88] and individuals undergoing allo-HCT [89], who are highly susceptible to invasive fungal infections, have increased levels of TCA and other primary bile acids, including glycocholic acid (GCA) and chenodeoxycholic acid (CDCA), which are also FXR ligands. Therefore, future studies to understand the role of primary bile acids on fungal colonization and its contribution to invasive fungal infections originating from the intestine is critical to gain insights into bile acid-mediated regulation of fungal infections in humans. The findings from our study spark several questions including: how does TCA impact the host defense system? What is the concentration-dependent effect of TCA on fungal colonization and dissemination? Do TCA and other primary bile acids, such as GCA and CDCA, affect other *Candida* pathogens, including *C. parapsilosis*, *Candida tropicalis*, and *Candida auris*? Can TCA alone promote fungal colonization and dissemination in the absence of microbiota? Can TCA alone cause intestinal barrier dysfunction in the absence of CA? In addition to the host and microbiota effect, TCA and (or) DCA may also have a direct effect on CA [26,90,91]. Given the significant alteration in bile acid levels during various therapies and diseases [30,31,32,33,34,35,36,37,38,39,40,41,42,43,44,45,46,47,48,49,50,51,52,92,93,94,95,96,97,98,99,100,101,102,103,104,105,106,107,108,109,110,111,112,113], understanding the direct and indirect mechanisms by which bile acids regulate fungal pathogens will provide a new approach to manipulate and control the fungal–bile communication system in favor of the host. In addition, CA in the gut also plays an active role in inflammatory bowel disease [102,114,115], the microbiota-gut-brain axis [116,117], and treating various pathogens, including *Clostridium difficile* [118,119]. Therefore, an in-depth understanding of the role of bile acid metabolites in affecting fungal colonization will form a strong platform to pursue therapies. Potential therapeutic strategies to prevent and treat invasive fungal infections and CA-associated intestinal diseases in humans could include the modulation of either the bile acids directly or bile-acid metabolizing commensal bacteria, or regulation of the host defense system through bile acid receptors.

## Data Availability

Not applicable.

## Supplementary Materials

The following are available online at www.mdpi.com/article/10.3390/jof7121030/s1, Table S1: Abundance of bile acids from cecal contents from individual mice samples; Figure S1: Bar graph showing the alpha diversity from cecal contents and mucosal scrapings from colon in the untreated and TCA treated mice groups infected with CA; Figures S2–S4: Heatmap showing the relative abundance of bacterial members (phylum, order and family) from cecal contents and mucosal scrapings from colon in the untreated and TCA treated mice groups infected with CA; Figure S5: TCA and DCA levels in the cecal contents from untreated and TCA treated mice infected with CA collected after 10-12 days of infection and treatment were shown here. 4–5 mice per group was used and the data represents mean ± SEM. Statistical significance was evaluated using the student’s *t* test. *p* values ≤ 0.05 (*) or ≤ 0.01 (**) were considered statistically significant.
