# Supplementary material for: Bile Acid Regulates the Colonization and Dissemination of Candida albicans from the Gastrointestinal Tract by Controlling Host Defense System and Microbiota
Source: J Fungi (Basel). 2021 Nov 30;7(12):1030. doi: 10.3390/jof7121030 (PMC8708873; doi:10.3390/jof7121030)
Supplement: Supplementary file 1 [file jof-07-01030-s001.zip › jof-1468224-supplementary/Supplementary Materials.pdf]

Supplementary Materials

Supplementary Figure S1. Bar graph showing the alpha diversity from cecal contents and mucosal scrapings from colon in the untreated and TCA treated mice groups infected with CA.

Supplementary Figures S2–S4. Heatmap showing the relative abundance of bacterial members (phylum, order and family) from cecal contents and mucosal scrapings from colon in the untreated and TCA treated mice groups infected with CA.

Supplementary Figure S5. TCA and DCA levels in the cecal contents from untreated and TCA treated mice infected with CA collected after 10-12 days of infection and treatment were shown here. 4-5 mice per group was used and the data represents mean ± SEM. Statistical significance was evaluated using the Student's t test. *p* values ≤0.05 (*) or ≤0.01 (**) were considered statistically significant.

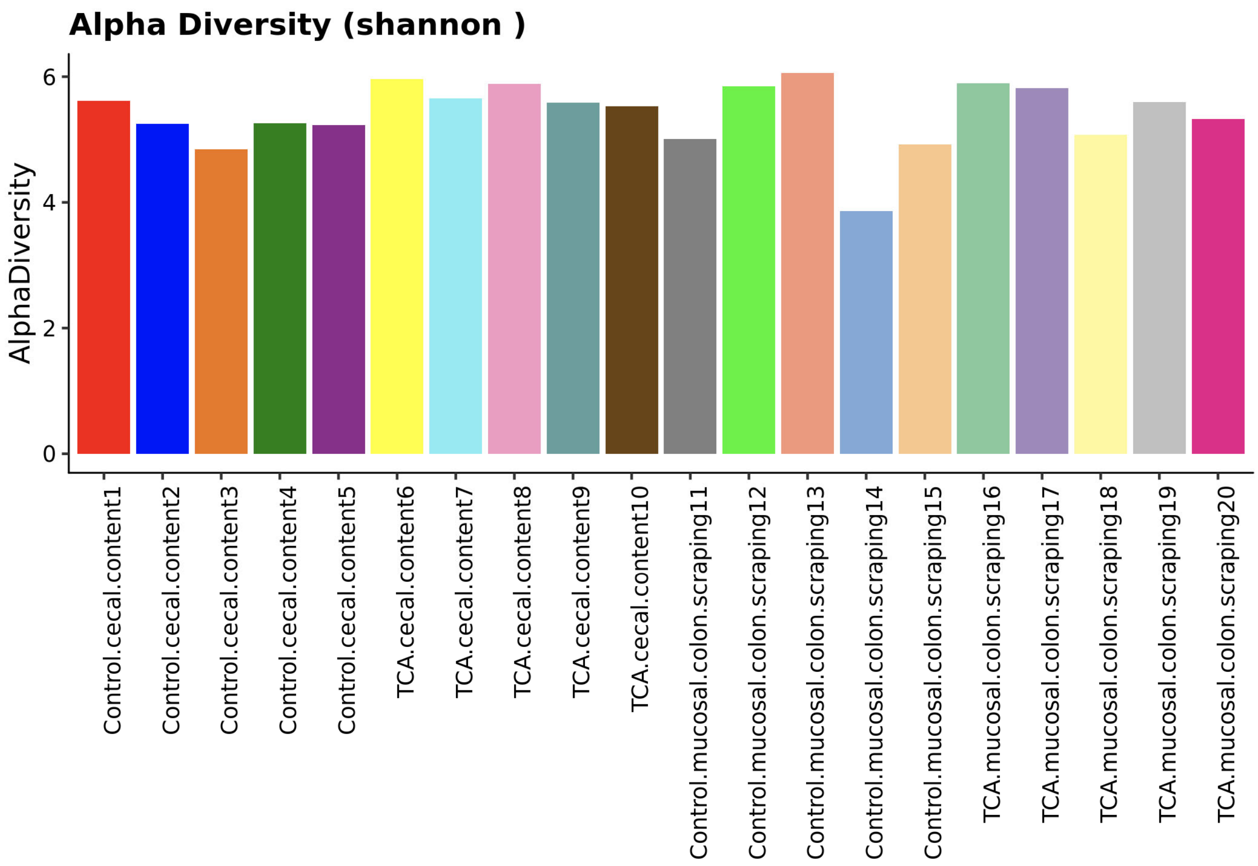

Supplementary Figure S1.

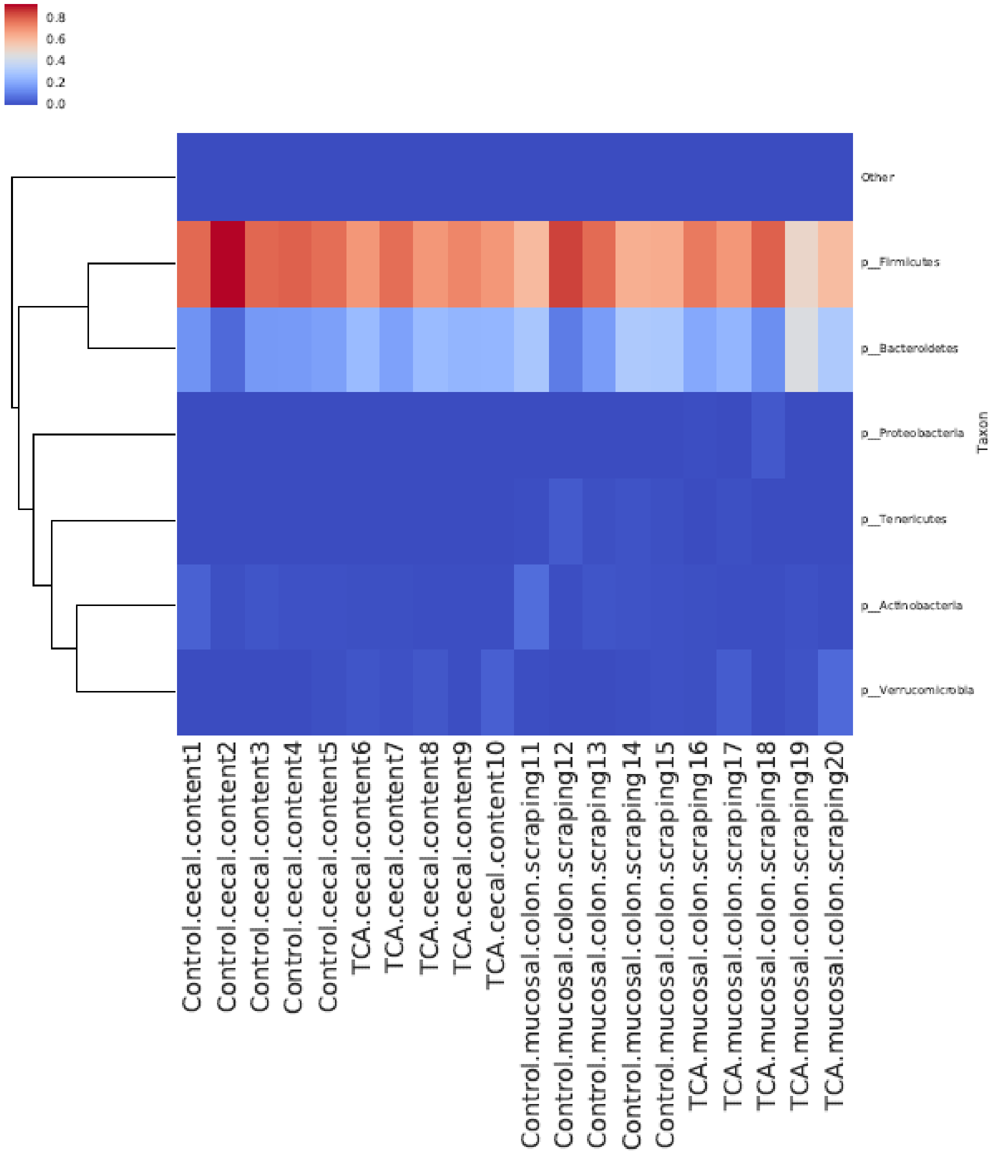

Supplementary Figure S2.

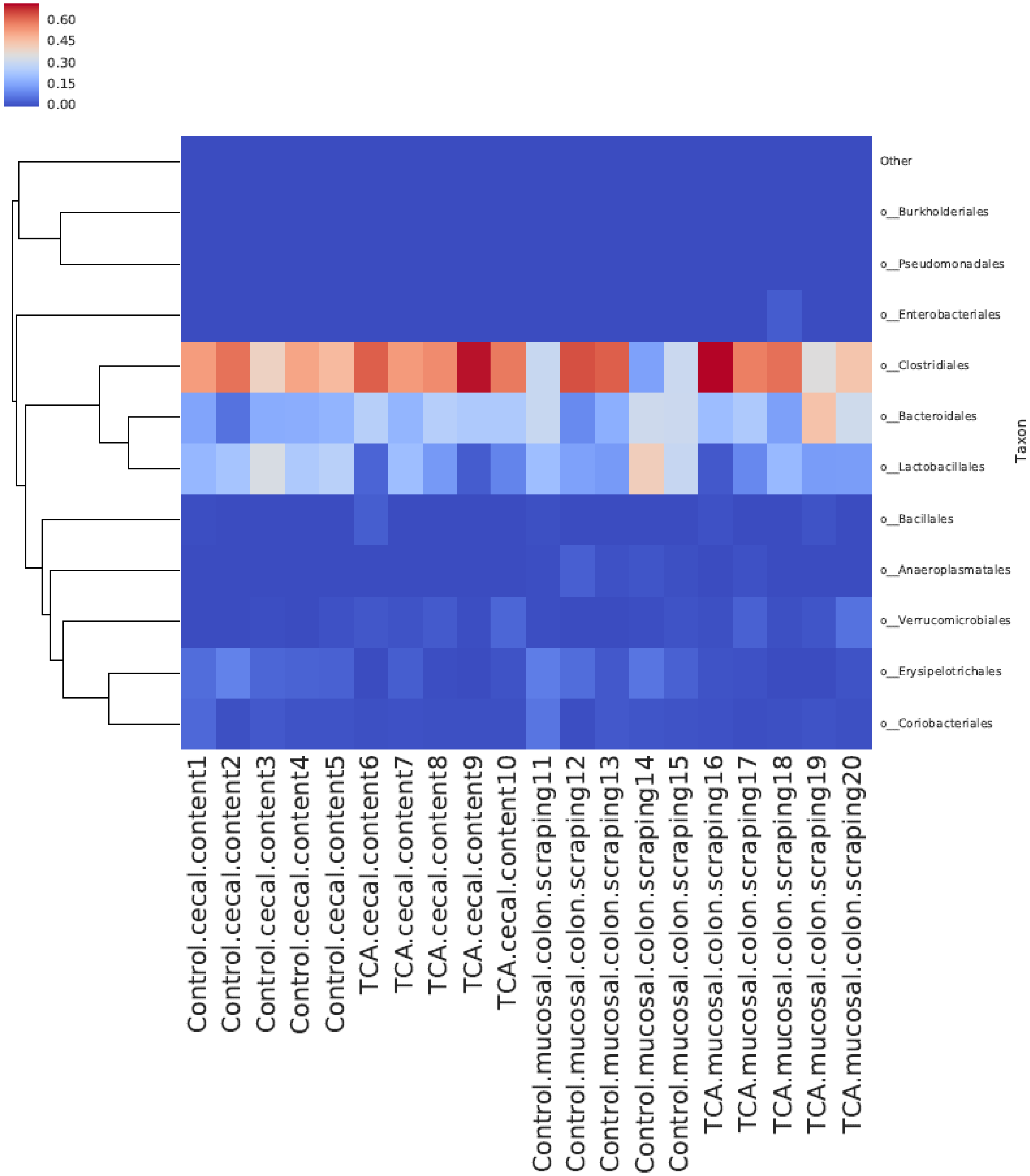

Supplementary Figure S3.

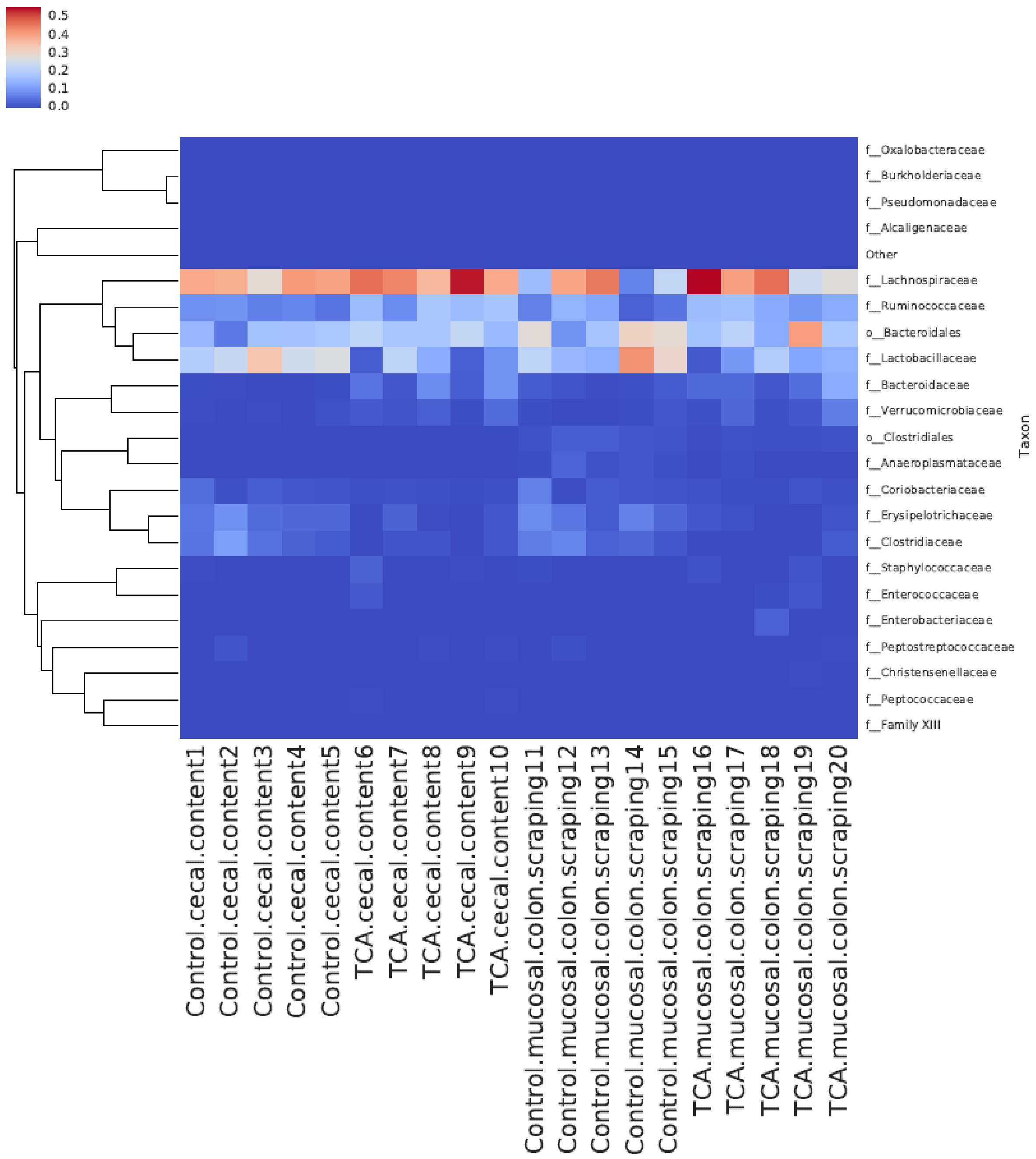

Supplementary Figure S4.

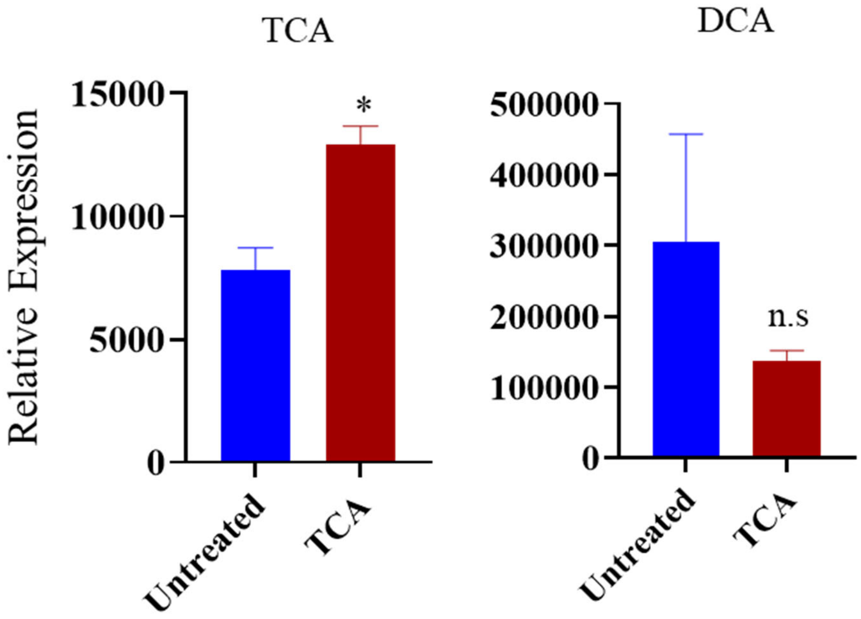

Supplementary Figure S5.
